# Predictors of skilled maternal health services utilizations: A case of rural women in Ethiopia

**DOI:** 10.1371/journal.pone.0246237

**Published:** 2021-02-19

**Authors:** Berhan Tsegaye, Elsabet Shudura, Amanuel Yoseph, Alemu Tamiso

**Affiliations:** 1 Department of Midwifery, Hawassa University Health Science College, Hawassa, Ethiopia; 2 Department of Sidama Region Health Department, Hawassa, Ethiopia; 3 Department of Public Health, Hawassa University Health Science College, Hawassa, Ethiopia; University of Mississippi Medical Center, UNITED STATES

## Abstract

**Background:**

Maternal health services are affected by complex factors from one setting to another. Consequently, health planners should prioritize different interventions and design appropriate programs to enhance maternal health services utilization. Results of prior studies are conflicting. Furthermore, only few studies were done from antenatal to postnatal continuum of care in Ethiopia.

**Objectives:**

This study aimed to assess prevalence and predictors of skilled maternal health services utilization at Dale-Wonsho health and demographic surveillance site of the Hawassa University, South Ethiopia, in 2019.

**Methods:**

A community based cross sectional study was conducted from January 1–30; 2019. A total of 682 women who gave birth in the last twelve months were selected by using a two stage sampling technique. Data were collected through face to face interview. Data were entered into Epidata version 3.1. Then, they were exported and analyzed by SPSS version 22. Bi-variable logistic regression analysis was done and variables with p-value less than 0.05 were considered as candidate for multivariable logistic regression analysis. Adjusted Odds Ratios (AOR) with 95% CI were computed, and p-value less than 0.01 was computed to determine the level of significance.

**Result:**

Prevalence of antenatal care, institutional delivery and postnatal care utilizations were 69.1%, 52.1% and 32.7% respectively. Educated women (AOR = 4.72, 95%CI,2.82,7.9), household training (AOR = 8.52,95%CI = 5.5,13.1), middle wealth quantile(AOR = 0.8,95%CI,0.4–0.7), being richest wealth quantile (AOR = 0.16;95%CI = 0.06,0.41) and pregnancy plan (AOR = 3.65,95%CI,1.67–8.0) were factors positively associated with antenatal care utilization. Husband education (AOR = 4.96,95CI,3.08–8.0), and antenatal care (AOR = 5.9; 95%CI,3.87,9.1) were factors associated with institutional delivery. Maternal education (AOR = 2.5,95CI,1.4–4.4), information about postnatal care service utilization (AOR = 3.6,95CI,2.1,6.2) and women autonomy(AOR = 6.1,95CI,3.8,9.7) were positively associated with postnatal care service.

**Conclusion:**

Prevalence of antenatal care, institutional delivery and postnatal care services were lower than the targeted plan. Policy makers should focus on capacity building of women both economically and academically. So, women should be more autonomous to utilize health services effectively. Moreover, awareness creation among women should be enhanced about maternal health service.

## Introduction

Globally, an estimated 303 million women died from complications related to pregnancy, childbirth and postpartum period in 2016 [1]. Nearly all of them were occurred in developing countries. From total maternal deaths in developing countries, more than half were happened in Sub-Saharan African countries [2]. Ethiopia is one of the top six Sub-Saharan countries with high maternal deaths. According to Ethiopian Demographic and Health Survey (EDHS) report, Maternal Mortality Ratio (MMR) was estimated as 420 per 100,000 live births in 2016 [3, 4]. In Ethiopia, more than 4 women from 5 of maternal died from complications amenable by lifesaving health interventions in health institutions [5]. Maternal deaths are occurred from pregnancy to postpartum continuum in different proportion: Pregnancy (25%), delivery (16%) and postpartum (61%) [3, 6].

Poor maternal health outcomes have been associated with underutilization of maternal health care services in most developing nations [4]. Hence, the world community focuses on maternal health nowadays [7]. Maternal health refers health of women during pregnancy, childbirth and postpartum period [8]. In Ethiopia, maternal health service utilization has been endorsed as the cost effective health approach to alleviate the burden of maternal deaths. It is one of the top priority agenda in reproductive health [9]. However, significant proportion of women were not still engaged fully in the continuum of maternal care with significant dropouts. For example, only 62% of pregnant women undergo at least one antenatal care visit, 26% of women gave birth in the health institution, and 16.5% of women received postnatal care in Ethiopia in 2016 [4]. Women with the same need should obtain the same health service. Consequently, the main concern of policies makers and health program planners is making maternal health service equally accessible everywhere [10]. Fail to access maternal health services is the predominant cause of high maternal morbidities and mortalities [11]. As a result, integrated community based maternal health services from pregnancy to postpartum period can improve maternal health outcomes [12, 13].

On the contrary, maternal health care service is affected with complex factors. For example, socio-demographic and reproductive factors are the frontline barriers for this problem [14–17]. According to behavioral model of heath service, the main influential factors were grouped under three main categories: predisposing, enabling and need related factors. These encompasses the individual and contextual dimensions [18, 19]. The context of underutilization of maternal health service varies from place to place. Hence, Compressive information on maternal health care utilization is a prerequisite to develop appropriate strategy to maximize its maternal health utilization at local national and international levels. Although many previous studies have been conducted in Ethiopia, results are still conflicting. For example, a study conducted in Enderta (Northern Ethiopia) indicates that more educated women delivered in health institution than less educated one [20]. However, another study in Holeta (Central Ethiopia) showed that more educated women were less likely to give birth in health institution [11]. Furthermore, according to a study married women utilize postnatal care less than unmarried women [21]. On the other hand, a study conducted in Jabitena Northwest Ethiopia indicated that married women are more likely to utilize postnatal care than their counterparts [22]. Besides, women of low parity utilized institutional delivery service more than their counterparts [11]. On the opposite side, women of low parity used institutional delivery service more than women of high parity [22]. Women of low monthly income utilize institutional delivery service less than women of high monthly income in Holeta, central Ethiopia [11]. On the contrary, a study conducted in South and North Ethiopia low income women utilize institutional delivery more than their counterparts [23]. But, results of studies were conflicting worldwide. For example, a study in Southern India indicated that religion has also shown association with maternal health service utilization significantly but not in some others [24]. In contrast, parity has been consistently shown to be negatively correlated with the use of skilled attendants from a study conducted in Thailand, Morocco, Kenya and Namibia [25–28]. Additionally, the above studies have reported positive association between economic status and use of institutional delivery service [24, 25]. Whereas, studies conducted in rural Guatemala and Tajistan have not found such an association between economic status and institutional delivery service [29, 30]. Furthermore, there are limited studies which assess the full skilled maternal health package. Skilled maternal health service utilizations are low coupled with inconsistent use of service from pregnancy to post-partum continuum. With this context, the country has less chance to achieve the 2030 national MMR reduction target of 70/100,000 live births. Therefore, this study aimed to assess prevalence and factors associated with maternal health care utilizations among women of reproductive age group who gave birth in the last 12 months at Hawassa University health and demographic surveillance system site, Dale and Wonsho district Health Demographic Surveillance Site (HDSS) of Hawassa university, South Ethiopia, in 2019.

## Methods and materials

### Study setting

This study was conducted at Dale-Wonsho HDSS (Health and Demographic Surveillance Site) of Ethiopia which is found in South region. It is controlled by Hawassa university. It was conducted from January 1–30; 2019. The study area is 330 km away from, Addis Ababa, capital city of Ethiopia. It is bordered in the North by Shabadino, in the south by Aleta-Wondo and Aleta chuko, in the east by Gorcha and in the west by Loka Abaya districts. There are a total of 43 Kebeles (the smallest administrative units in Ethiopia) in the district. Among these, 39 Kebeles are rural and 4 were urban Kebeles. According to 2015 population projection estimation, there were a total of 365,652 residents in the study area. From these, only 61,430 were women of reproductive age group (15–49 years) [31]. Maternal health services are provided by a total of 68 public health institutions in the study area. These include the following:1 general hospital and 15 health centres. Furthermore, there were about a total of 1259 health providers.

### Study design and population

A community based analytical cross-sectional study was conducted from January 1–30;2019. The source population were women of the reproductive age group who gave at least one live birth in the last twelve months preceding survey. Furthermore, the study population were women of reproductive age group who gave at least one live birth in the last twelve months preceding survey, selected for this study and presented during data collection period. Women who lived less than six months in the study area, and women who were unable to communicate were excluded from this study.

### Sample size calculation and sampling procedure

Sample size was calculated by using single population proportion formula in consideration of the following assumptions: Prevalence of antenatal (74.3%), skilled delivery service (28.7%) and postnatal (22.6%) [32], 95% confidence interval, Z score (1.96), and margin of error (d) 5%. Thus, sample sizes for the first objective were computed using the following formula: n = Z^2^(1-α/2) P(1-P)/D^2^. Sample sizes were computed for antenatal, skilled delivery service and postnatal care utilization as 294, 314 and 269 respectively.

We considered the design effect of 2 to minimize bias arising from not using simple random sampling technique. Furthermore, we tried to minimize errors arising from chance of noncompliance by adding 10% of the total sample sizes. In general, the total sample sizes for prevalence were calculated as: 647, 692 and 591 for antenatal care, skilled delivery care and postnatal care service uptake respectively. The sample sizes for associated factors (second objective) were calculated using Stat Cal command of epi-info software package from the same study in which sample size for prevalence was calculated. Finally, the final sample size was taken as 692 which is calculate sample size to achieve the objectives of the current study. A two stage sampling procedure was used to select the study participants. Firstly, 12 Kebeles were selected using simple random sampling which account for 20–30% of the total Kebeles in the district. There are a total of 16,800 households Kebeles in the study area.

By using HDSS data base as a sampling frame, we have identified households which contains women of the reproductive age group who gave live birth in last twelve months. Then, we applied proportional allocation to draw the study participants from each selected Kebele. Secondly, we select the study participants from each Kebele using simple random sampling. If woman was absent from the household during consecutive visits, she was regarded as non-response. For households with more than one eligible women, the index woman was selected by simple random sampling using lottery method. Neighbors were asked whether an eligible woman was present or not in the house. Revisits of two to three times were made in case where eligible respondents were not available at the time of the survey. Finally, they were considered as non-respondents.

### Measurement

The dependent variables were skilled maternal health services utilization. These include the following three consecutive cares: Antenatal care service, skilled delivery care service and postnatal care service utilization. Each variable is binary outcome. Each variable was labeled as ‘0’ for non-utilization and ‘1’ for utilization of the respective service.

Independent variables were socio-demographic and reproductive health variables in this study. These explanatory variables were selected from various literatures [33–36]. The socio-demographic variables included the following charactestics: Age, educational status, occupational status, wealth index, family size, marital status and religion. Principal component analysis was done to determine wealth index. Wealth index was constructed using principal components analysis on household asset data. Individuals were classified into five wealth quintiles (poorest, poorer, medium, richer and richest). Variables included in the wealth index were ownership of selected household assets, size of agricultural land, quantity of livestock and house construction materials [37]. It was built in three consecutive steps for maximum adaptability of urban and rural areas. a subset of indicators was used to create wealth score of households in both areas in the first step. Separate factor scores were constructed for each household in both areas using specific indicators. In the third step, a nationally combined wealth index was created by combined specific scores for separate area through adjusting of area specific score with regression on a common factors score [38].

Furthermore, reproductive variables included: Antenatal care visits, time of initiating antenatal care visit, tetanus toxoid vaccine utilization, problem faced during pregnancy, reasons for antenatal care visit, place of delivery, type of health institutions visited, place of delivery, type of health providers attend, post-natal checkup, frequency of postnatal checkup and time to start postnatal checkup.

### Operational definitions

#### Skilled attendants

Health professionals who can identify, treat and refer women with many obstetrics complications at pregnancy, delivery and postnatal period timely. These include: Doctors, midwives, nurses, health officers and emergency obstetrics and surgeons.

#### Proportion of antenatal care

Is defined as the proportion of complex of interventions that a pregnant woman receives from organized health care services by skilled delivery attendant at least one visit in the recent pregnancy.

#### Women’s autonomy

A more autonomous woman is a woman who can decide on health care spending alone with her husband. If the decision of health care spending is controlled by other than the women herself, it is considered as non-autonomous.

#### Proportion of safe delivery service utilization

Is the proportion of women who receive delivery care provided in the health institution by skilled health providers during a study participant’s recent birth.

#### Proportion of postnatal care

Is defined as the proportion of women who receive care for them and their newborn given to immediately after the birth of the placenta and for the first 42 days of life [39].

### Data collection

Data were collected using the structured and pretested questionnaire via face to face interview at the participant’s home. The questionnaire was adapted from Ethiopian demographic health survey tool. It was constructed in English and then translated into local language (Sidama-afoo) and back to English to keep consistency. Inconsistent and inaccurate data were readjusted accordingly. Data were collected 8 HDSS data collectors. Furthermore, two public health experts supervised the data collection process. Training was given for data collectors and supervisors for two consecutive days. They were trained about aim of study, procedures, collection techniques, art of interviewing and ways of collecting the data. Pre-testing was done on 5% of samples on Kebeles out of the study area. Each questionnaire was checked for completeness each day after data collection by the supervisors. Finally, feedback was given for data collectors.

### Data analysis

Data were entered, cleaned, coded into Epidata software version 4.2. Then, data were exported and analyzed using SPSS 22. We utilized frequencies, means and percentages to summarize the descriptive statistics. Tables and graphs were used for the data presentation. Bi-variable logistic regression analysis was done to identify variables associated with skilled maternal health service utilizations. Consequently, variables whose p-value less than 0.05 were considered as candidate variables for multivariable logistic regression analysis. In multivariable logistic regression analysis, variables with p-value less than 0.01 and adjusted odd ratios with 95% confidence interval were reported as statistically significant variables. Model fitness was checked by using Hosmer-Lemeshow test.

### Ethical considerations

First, ethical clearance was obtained from institutional review board of Hawassa university college of medicine and health science. Second, a letter of support was obtained from Sidama zone health department. Third, we obtained administrative permissions from every kebele. Privacy and confidentiality of study participants was assured with a maximum effort. Then, each participant signed informed written consent. Finally, parents or guardians who live together provided written consent on behalf of study participants below 18 years old.

## Result

### Socio-demographic characteristics of the study participants

Table 1 of this study indicated the socio-demographic characteristics of the study participants. From a total of 692 study participants recruited for this study, only 682 study participants gave full response making response rate of 98.67%.

**Table 1:** Socio-demographic characteristics of study participants(n = 682) at Hawassa HDSS, South Ethiopia, in 2019.

Variables	Categories	Frequency (%)
Age	15–24	213 (31.2%)
	25–34	329 (48.2%)
	35–49	140 (20.5%)
Ethnicity	Sidama	661 (96.9%)
	Amhara	19 (2.8%)
	Guarage	2 (0.3%)
Religions	Protestant	632 (92.7%)
	Orthodox	17 (2.5%)
	Muslim	18 (2.6%)
	Catholic	15 (2.2%)
Marital status	Married	634 (93%)
	Cohabiting	4 (0.6%)
	Divorced	38 (5.5%)
	Widowed	6 (0.9%)
Maternal educational status	No formal education	132 (19.4%)
	Educated	550 (80.6%)
Husband educational status	No formal education	141 (20.7%)
	Educated	541 (79.3%)
Occupation of the mother	Housewife	631 (92.5%)
	Government employer	6 (0.9%)
	Merchant	39 (5.7%)
Occupation of the husband	Farmer	497 (72.9%)
	Government employer	45 (6.6%)
	Merchant	112 (16.4%)
	Others	28 (4.1%)
Wealth Index	Poorest	143 (21%)
	Poor	125 (18.3%)
	Middle	156 (22.9%)
	Rich	75 (11%)
	Richest	183 (26.8%)
Social media access	Yes	400 (58.7%)
	No	282 (41.3%)

According to the report of this study, nearly half (48.2%) of the study participants were in the age range of 25–34 years. Most 632(93%) of the study participants were follower of protestant religion. Furthermore, majorities 634(93%) of the study participants were married. Majorities, 214(60.1%) of study participants were merchants. Furthermore, one-hundred- thirty-two (19.4%) of women and one-hundred-forty-one (20.7%) of their partners never attended formal education. Approximately one in four women was in richest wealth index. From the total of study participants, 400 (58.7%) of the study participants had access to mass media.

### Reproductive health characteristics of the study participants

Table 2 indicated the reproductive health charactestics of the study participants. According to this study report, majorities 471(69.1%) of the study participants had antenatal care visits for their most recent birth. Most (79.6%) of the study participants were in the age range of 20–34 years. More than half 459 (67.3%) of the study participants planned their pregnancy. Approximately 1 from 2 women got pregnancy of more than five times. Moreover, two-third of the study participants delivered five and above times. More than half 404 (59.3%) of the study participants gave birth 1–4 times. Half 236 (50.1%) of the study participants started antenatal care in the last three months. Nearly half (47.9%) participants delivered in their home. Majorities (67.3%) of the study participants did not attend postnatal care service.

**Table 2:** Reproductive health characteristics of women of reproductive age group (n = 682) in Hawassa HDSS, South Ethiopia, in 2019.

Variable	Categories	Frequency (%)
Antenatal care visit	Yes	471 (69.1%)
	No	211 (30.9%)
Age at last pregnancy	15–19 years	38 (5.6%)
	20–34 years	543 (79.6%)
	35–49 years	101 (14.8%)
pregnancy status	Planned	459 (67.3%)
	Unplanned	223 (32.7%)
Gravidity	1–4	446 (65.4%)
	5 and above	236 (34.6%)
Parity	1–4	404 (59.3%)
	5 and above	278 (40.7%)
Reason for not using antenatal care (n = 211)	No knowledge	36 (17.1%)
	Being in a good state	83 (39.3%)
	Far from my house	48 (22.7%)
	Too busy	44 (20.9%)
Time started antenatal care use	Less than 3months	78 (16.6%)
	3–6 months	157 (33.3%)
	More than 6 months	236 (50.1%)
Place of delivery	Health facility	355 (52.1%)
	Home	327 (47.9%)
Types of health facilities visited (n = 355)	Hospital	132 (37.2%)
	Health center	207 (58.3%)
	Health post	16 (4.5%)
Reason for non-utilization of institutional delivery (n = 327)	Dislike health facility	86 (26.3%)
	Delay of ambulance	119 (36.4%)
	No road access	122 (37.3%)
Health provider attended delivery (n = 355)	Doctors	80 (16.5%)
	Midwifes	71 (15.3%)
	Nurse	340 (68.3%)
	Others*	7 (1%)
Postnatal check up	Yes	223 (32.7%)
	No	459 (67.3%)
Main reason initiated postnatal care use (n = 223)	Health problem	48 (20.9%)
	For regular checkup	124 (55.6%)
	Immunization service	51 (22.9%)
Time of PNC checkup (n = 223)	Immediately after delivery	97 (43.5%)
	Within 24 hours	73 (32.7%)
	More than 2 days	53 (23.8%)

**Key:** Others* **=** health officers, emergency obstetrics and surgeon.

### Prevalence of skilled maternal health services utilization

**Fig 1:**
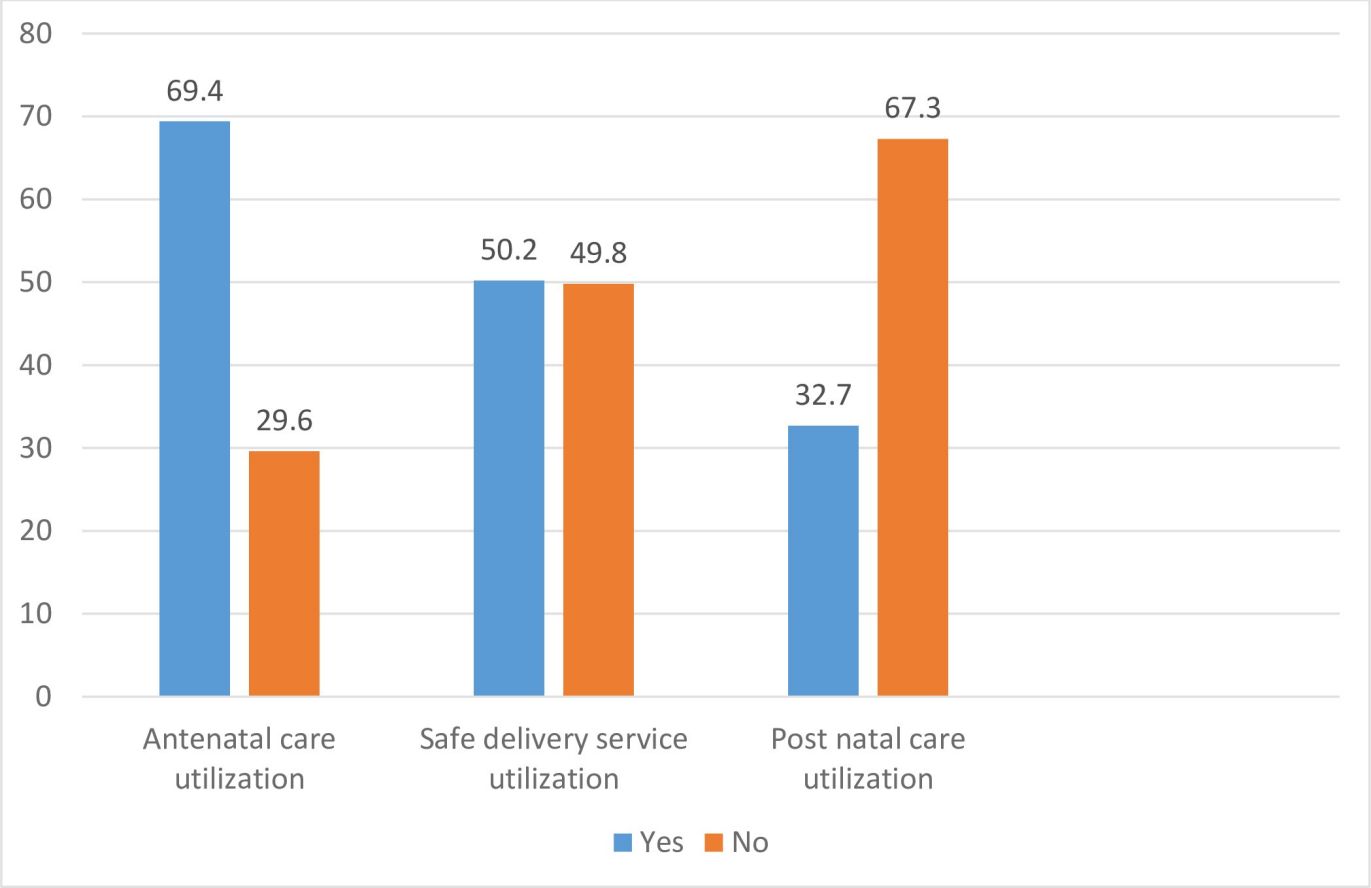
Proportion of skilled maternal health services among women of reproductive age group (n = 682) in South Ethiopia, in 2019.

Fig 1 showed the prevalence of skilled maternal health services utilization. The prevalence of antenatal care, skilled delivery care service and postnatal care utilization among women of reproductive age group were 69.1%, 52.1% and 32.7% respectively.

Among the total study participants, eighty-three (39.3%) of the study participants did not utilize antenatal care service as they perceived no need of care due to their well health condition. The main reasons for non-utilization of institutional delivery were lack of road access 122 (37.3%), and ambulance delay 119 (36.4%) in the current study. Half (50.1%) of the study participants started antenatal care more than six months. Among births attended in the health facilities, most 207 (58.3%) of deliveries were attended in health center. From these, majorities 340 (68.3%) of deliveries were attended by nurses. Among study participants, more than half (55%) of them came for regular checkup. Most 97 (43.5%) of women started postnatal care immediately after birth.

### Factors associated with skilled maternal health service utilization

Table 3 showed that the result of bi-variable and multi-variable logistic regression analysis of antenatal care utilization. Hence, eight variables were associated with antenatal care utilization statistically. These variables were: Wealth index, maternal education, model household training, road access, plan of pregnancy, access to information on Antenatal care and counseling about pregnancy danger sign. Among these factors: wealth index, maternal education, model household training and having pregnancy plan were factors associated with antenatal care utilization in the multi-variable logistic regression. Accordingly, the odd of antenatal care utilization among women who planed current pregnancy was higher odds than their counterparts (AOR = 3.65; 95% CI = 1.6,8.0). The chance of antenatal care utilization was higher among women who had a formal education than their counterparts (AOR = 4.72, 95% CI = 2.82,7.9). In addition, compared to women who did not receive model household training, women were received model household training were more likely to use antenatal care service (AOR = 8.52; 95% CI = 5.5,13.1). The odds of women in richest wealth quantile was lower than women in poorest quantile by antenatal care utilization (AOR = 0.16; 95%CI = 0.06,0.41). However, the odds of utilization of antenatal care was increased by 4.9 times for women who had educated husbands (AOR = 4.96, 95% CI = 3.08,8.0) than their counterparts.

**Table 3:** Predictors of antenatal care utilization among women of reproductive age group (n = 682) in Hawassa HDSS, South Ethiopia, in 2019.

Variables	Category	Antenatal utilization		COR	AOR
		Yes	No		
Wealth index	Poorest	84	59	Ref.	Ref.
	Poor	67	58	1.23 (0.75, 2.0)	0.94 (0.53, 1.64)
	Middle	136	20	0.2 (0.12, 0.37)*	0.09 (0.04, 0.19)**
	Richer	48	27	0.8 (0.45, 1.42)	0.47 (0.20, 1.08)
	Richest	136	47	0.5(0.30, 0.78)*	0.17 (0.07, 0.42)**
Formal maternal education	Yes	413	137	3.85 (2.59, 5.7)*	4.72 (2.82, 7.90)**
	No	58	74	Ref.	Ref.
Model household Training	Yes	333	55	6.84(4.75, 9.86)*	8.52(5.52,13.15)**
	No	138	156	Ref.	Ref.
Road accessibility	Yes	406	196	0.48(0.27, 0.86)*	0.57 (0.27, 1.22)
	No	65	15	Ref.	Ref.
Media access	Yes	259	141	0.61(0.43, 0.85)*	0.69 (0.36, 1.34)
	No	212	70	Ref.	Ref.
Current pregnancy planned	Yes	448	180	3.36 (1.90,5.91)*	3.65 (1.67, 8.01)**
	No	23	31	Ref.	Ref.
Information about antenatal care	Yes	427	170	2.34(1.47, 3.71)*	1.43 (0.67, 3.05)
	No	44	41	Ref.	Ref.
Counseled about danger sign	Yes	361	145	1.49(1.04, 2.14)*	1.56 (0.86, 2.85)
	No	110	66	Ref.	Ref.

**Key:** Ref: Reference categories

*: P-value < 0.05 in binary logistic regression analysis

**: P-value < 0.01 in multivariable logistic regression analysis.

According to Table 4 report, independent variables associated with institutional delivery service utilization in bi-variable logistic regression were: Age of women, the presence of maternal formal education, husband education, model household training and antenatal care utilization. However, only husband education and ANC utilization showed significant association. Based on the result, the odds of safe delivery among women who had antenatal care visit was 5.9 times higher than their counterparts (AOR = 5.9; 95% CI = 3.8,9.2) and the odds of women with uneducated husband were 80% less likely to conduct safe for delivery (AOR = 0.20; 95%CI,0.13, 0.32).

**Table 4:** Predictors of skilled delivery care utilization among women of reproductive age group (n = 682) in Hawassa HDSS, South Ethiopia, in 2019.

Variables	Category	Skilled delivery service utilization		COR	AOR
		Yes	No		
Age of women	15–24 years	119	94	Ref.	Ref.
	25–34 years	175	154	1.11 (0.71, 1.36)	0.84 (0.55, 1.28)
	35–49 years	61	79	1.64 (1.01, 2.98)*	0.77 (0.44, 1.38)
Formal maternal education	Yes	48	84	Ref.	Ref.
	No	307	243	0.45 (0.31, 0.67)*	0.87 (0.53, 1.42)
Model household Training	Yes	244	144	2.79 (2.04, 3.82)*	1.43 (0.98, 2.08)
	No	111	183	Ref.	Ref.
Husband education	Yes	31	110	Ref.	Ref.
	No	324	217	0.19 (0.12, 0.29)*	0.20 (0.13, 0.32)**
Antenatal care utilization	Yes	309	162	6.84(4.68, 9.98)*	5.96 (3.88, 9.18)**
	No	46	165	Ref.	Ref.

**KEY:** Ref: Indicates the reference categories

*: P-value < 0.05 in binary logistic regression

**, P-value <0.01, multiple logistic regression.

Based on Table 5 result, crude odds ratios showed that five variables were associated with postnatal care utilization significantly. Among these, only three variables were associated significantly with postnatal care utilization. These were maternal formal education, information about postnatal care and women autonomy in decision making were statistically associated with postnatal care in the final model. These association is illustrated as follows: The likelihood of postnatal care utilization was higher among educated women than their counterparts (AOR = 2.6; 95% CI = 1.4,4.4). The odd of postnatal care utilization was 3.6 times higher among women who had information about postnatal care utilization than their counterparts (AOR = 3.6; 95% CI = 2.1,6.2). Women were autonomous in decision making process on health related matters had more chance of utilizing postnatal care service than their counterparts (AOR = 6.1, 95% CI = 3.8,9.7).

**Table 5:** Predictors of postnatal care utilization among women of reproductive age group (n = 682) in Hawassa University Health and Demographic Surveillance Site, South Ethiopia, in 2019.

Variables	Category	Postnatal care use		COR	AOR
		Yes	No		
Formal maternal education	Yes	20	112	Ref.	Ref.
	No	203	347	3.28 (1.98, 5.46)*	2.57 (1.48, 4.44)**
Current pregnancy planned	Yes	214	414	2.58 (1.24, 5.39)*	1.69 (0.74, 3.84)
	No	9	45	Ref.	Ref.
Information about post-natal care	Yes	201	297	5.22 (3.20, 8.51)*	3.66 (2.18, 6.14)**
	No	21	162	Ref.	Ref.
Place of delivery	Yes	131	224	1.49 (1.08, 2.06)*	1.23 (0.85, 1.76)
	No	92	235	Ref.	Ref.
Autonomy of women	Myself	197	236	7.16(4.57, 11.2)*	6.13 (3.86, 9.73)**
	Others	26	223	Ref.	Ref.

**KEY:** Ref.: Indicates the reference categories

*: Indicates significant association (P-value < 0.05)

**, indicate the highly significant association (P-value <0.01).

## Discussion

Poor uptake of maternal health service is one of the leading cause of maternal morbidity and mortality in the world [40]. This study is the one of the first predominant study which can resolve the preexisting conflict among findings of different studies about skilled maternal care service utilization. Leaving these conflicts unsolved at individual level can lead to miss the target point of intervention during policy making and program design. Moreover, this study provides adequate picture of the problem of poor uptake of skilled maternal care service as continuum of care from pregnancy to postpartum period. Hence, this study explored determinants of skilled maternal care mainly at a rural community. Primary health services (skilled maternal care services) inequality predominantly existed in the rural population for many years in Ethiopia.

Prevalence of antenatal care follow-up, skilled delivery care and postnatal care service utilization were estimated as 69.1%, 52.1% and 32.7% respectively. According to Mini-EDHS 2019 report, antenatal care service utilization was estimated as 69.4%(95%CI,67.6%,71.0%) during pregnancy, skilled delivery care was reported as 50.2%(95%CI,48.7%,51.7%), and post-natal care service was measured as 32% (95%CI,29.7%,34.3%) in South Nation Nationalities and Peoples Regional states of Ethiopia [41]. Based on these finding, compared with the regional proportion of skilled maternal health service, antenatal care and postnatal care utilization in this study are in line with regional proportions. However, skilled delivery service utilization was slightly higher than the regional proportion. These findings clearly indicate that maternal health service utilizations are improve active involvement of health extension workers and their community mobilization of the health development army in the rural areas. Previous evidence proved this argument that health extension workers did not achieve significant change in antenatal and postnatal care service as safe delivery service in Ethiopia [23]. On the contrary, the probable justification of increased proportion of skilled delivery care service in this study might be due to better quality antenatal care service at pregnancy by health extension workers than other parts of regions. A previous study consolidated this justification than women who had frequent visits by health extension workers were more likely to visits health institutions in Ethiopia [42]. Moreover, antenatal care service utilization was higher in this study as compared with findings of studies in other parts of Ethiopia: Debretabor, South Gondar (35.5%), East Wollega (14.4%), Dejen, East Gojjam(12%) and East Hararge (38.3%) [43–46]. This could be due to variation in study population groups, study setting and health service infrastructure. This argument was supported with an evidence proved that expansion of primary health facilities increased maternal and child health program interventions [47]. Furthermore, some cultural practices might encourage home treatment for complications during pregnancy in the study area than above study areas. prevalence of skilled delivery service uptake is consistent with finding of study done in Woldia, Northwest Ethiopia (48.3%) [48]. The possible explanation might be due to similar study population, study setting and socio-economic status of study participants. On the contrary, the this finding is more than finding of studies done in Butajera, South Ethiopia (15.7%), Banja, North West Ethiopia (37.9%) and Enderta, North Ethiopia (25%) [49–51]. The possible explanation for this discrepancy could be due to better awareness of women and strong health provider’s commitment towards birth preparedness and complication readiness plan during pregnancy in this study. Furthermore, home delivery is more cultural and religious practice in the North Ethiopia than South Ethiopia. To the opposite side, this finding is lower than findings of studies carried out in Holeta, Central Ethiopia (61.6%) and Sodo, South Ethiopia (62.2%) [52, 53]. This might be due to fact that the study participants in current study were rural dwellers than others. Consequently, rural women could not be autonomous in decision making power about their health, there might be lack of access to information and inadequate infrastructure construction in the study area. Prevalence of postnatal care utilization is higher than findings of other studies in Ethiopia: Jabitena, North West Ethiopia (20.2%), Haromaya, Eastern Ethiopia (22.6%) and North Shoa, Central Ethiopia (28.4%) [4, 16, 54, 55]. This might be due to the efforts exerted by community health workers to increase women’s awareness on the benefit of postnatal care utilization in the current study setting than others. Previous study proved that women who had better knowledge about danger sign during postpartum period were better utilized postnatal care service than their counter parts [56]. The absence of cultural belief by community members who perceives movement outside of home could exposed women for evil spirit might increase postnatal care use by restricting movement of women after delivery in the current study area than others. On the contrary, the this finding is lower than other similar studies in the Enderta, North Ethiopia (49.7%), South Ethiopia (37.2%) and Assella (72.7%) [57–59]. These discrepancies might be due to difference in socio-demographic characteristics and health seeking behaviors of women. Moreover, cultural practice and extent of urbanization among study settings might influence postnatal care service utilization.

Educational status, model household training, wealth quintile and pregnancy plan were important factors associated with antenatal care utilization in this study. Specifically, women who planned their pregnancy plan were utilized antenatal care service more than their counterparts. This finding was in line with many studies in Ethiopia. For example, a study done in Debretabor town, Wonberma and Yem woreda and South Africa [43, 60–62]. The authors argued that women who plan to have a child have better awareness of their health. As a result, they can make all the necessary arrangement to use antenatal care service effectively. This study proved that wealth index was critical determinant of antenatal care service utilization. As we go down from richest to poorest wealth index category, women had low chance of using antenatal care. Similar findings were also reported from studies conducted in the Nigeria and Ethiopia [4, 63]. The possible rational might be due to direct and indirect costs for service utilization. Even though antenatal care is free of charge currently in Ethiopia, there are indirect costs of transportation, accommodation during staying in towns, food costs and some medicine costs. An evidence has been argued that mother from poor communities or scarce resources may have difficulties to pay for the healthcare costs [64]. In addition, rich women have sufficient purchasing power to healthcare and materials of information. Previous researchers argued that rich women had tend to have personal control over resource, and being autonomous in decision making process than poor women [65]. As do many other studies, this study also indicates that educated women were more likely to utilize antenatal care than their counterparts [43, 44, 64]. The possible rational might be that as the women educated more, they tend to have good health seeking behavior, more autonomous and economically secured to utilize antenatal care service. Furthermore, education make women more positive thinker and confident.

Lack of husband education had influential effect on institutional delivery utilization in the current study. However, other studies which were done in Bangladesh and Benishangul-Gumuz of Ethiopia have disproved this hypothesis [66, 67]. The possible disparities might arise from the fact that husbands are more conservative towards their cultural practice of home delivery and women are not autonomous both in Bangladesh and Benishangul Gumuz of Ethiopia regardless of their educational status. This might indicate that education by itself might not change husbands’ behavior except massive work is done to change on their attitude. Utilization of at least one antenatal care visit increased institutional delivery service utilization in this study. Similar finding have been also reported in Benishangul-Gumuz region, Wukro and Butajera, Sodo South region and Munisa Oromia region in Ethiopia [51, 53, 66, 68]. This finding may suggest that women who have antenatal care visit prepared for birth and complication and anticipate its outcome. Previous studies also documented that birth preparedness and complication readiness plan has associated positively with institutional delivery place [69]. Maternal education was found to improve postnatal care utilization in this study. This finding is in agreement earlier works [55, 70–72]. This can be elaborated by the notion that education is an important factor in empowering women in decision making power towards postnatal care utilization, increasing awareness of fundamental health care services, and increase problem detection power of women and minimize economic dependence through employment. Consequently, women can be directed to positive health seeking behavior. This study also indicates that information access about service influenced post-natal care service positively. This finding was in agreement with findings of Dembecha, Northwest Ethiopia [73], Nigeria and Nepal [71, 72]. The observed similarities might be that informed women have good knowledge and skill of maternal health care service utilization. Finally, autonomous in decision making power was another critical factor associated with uptake of post-natal care service. Women who were more autonomous in decision making power utilized postnatal care more than their counterparts. This finding is consistent with other similar studies [74–76]. The possible reason might be due to the fact that women can increase bargaining power with their husbands on postnatal care service utilization as they become more autonomous.

### Limitation

This study has several strengths. First, the rural and community based nature of the study can indicate accurate picture of the problem. Second, using simple random sampling technique, adequate sample size and standard data collection tool could yield reliable estimates. Third, each hypothesis was tested for demand side individual level variables with complete picture of the problem for maternal health care services from pregnancy to postpartum continuum.

On the contrary, the current study had few limitations. For example, recalling bias might be the source of bias. To reduce the recall bias, for instance, only women who gave birth in the last one year were selected. Furthermore, the current study did not include supply side factors of maternal health service utilization. Therefore, further studies should be conducted to assess the health institutional factors of maternal health service.

## Conclusion

In general, maternal health care services are still unsatisfactory in this study compared to the target plan of local and national proportions. Maternal education, model household training, higher wealth quantile and having pregnancy plan have increased antenatal care use. Husband education and antenatal care service utilization were positively associated with skilled delivery care. Furthermore, information about postnatal care, maternal education and more autonomous status of women were associated with increased odd of postnatal care utilization. This finding warrants policymakers to formulate strategies to enhance awareness about maternal health services utilization. Ethiopia government should enhance socio-economic status of women. Furthermore, community maternal health services should be improved from pregnancy to postpartum period continuum. Moreover, women should plan their pregnancy; otherwise, use family planning method consistently.

## Supporting information

S1 File Dataset. (SAV)
